# Repertory of the Back

**Published:** 1899-10

**Authors:** E. H. Wilsey

**Affiliations:** Parkersburg, W. Va.


### REPERTORY OF THE BACK.

E. H. Wilsey, M. D., Parkersburg, W. Va.

(Continued from Aug. number, page 378.)

SACRUM.

**Support**, often placed her hand on sacrum to support it and complained of a pain in sacro-iliac synchondrosis, Oleum-jec.

**Suppurating**, a spot in sacrum feels as if suppurating, worse by touch, Colch.

**Tearing**, and burning in sacrum and hips afternoon and night, Mag-m.

— in sacrum, kidneys, and back, especially near spine, *Lyco.*

— in sacrum extending to occiput left half of brain and inflamed eyes and bloated cheeks, Ledum.

— intermittent tearing in sacrum after rising from stooping down; while standing still it quietly draws in jerks, Phos-ac.

— a fine tearing pain in sacrum while sitting, passing from right to left upward, Spong.

— cutting, tearing low down at both sides of sacrum, Mez.

— and sharp shooting in sacrum on moving, Dig.

— pressure in sacrum, Carbo-v.

— and shooting in sacral bones, Zinc.

— fine tearing, drawing from middle of sacrum toward lumbar vertebræ, Mur-ac.

— in sacrum transversely while sitting up straight, Lyco.

**Tenderness,** extreme over sacrum, Lob.

**Tension** and sensation of weakness in sacrum when sitting, with tension in head, Zinc.

— painful tension in sacrum so that he cannot breathe deeply, Nit-a.

— piercing pain and tension in sacrum, Sul.

— in left sacral region and hip, Sul.

**Tensive,** cutting thrusts in sacrum, worse bending forward, with tensive pain, Sambucus.

### SACRUM.

**Tensive**, pain from sacrum across left hip on least motion, interfering with walking, Sarsa.
— pain near left side of top of sacrum when walking, Sumbul.
— pain in sacrum, Thuja.
— pain at the slightest movement extending from sacrum over hip, impeding walking, Sarsa.
— pain and stiffness in sacrum, Carbo-v.
— violent tensive pain in sacrum, Caustic.
— pain in sacrum, worse evening, so that he could not raise from his seat nor bend backward, *Bar-c.*
**Throbbing**, a threatening throbbing in lower part of sacrum, Bar-c.
— in sacral region in evening, Tabac.
— in left side of sacrum, Sumbul.
— severe in sacrum, *Nat-m.*
— in sacrum, Graph., Ign.
— aching, beating, throbbing pain in sacro-lumbar region, Sil.
— frequently in sacrum, Caustic.
— a steady throbbing pain in sacrum in a small spot like a gathering, most felt at night, and hinders sleep, violent aching pain, better in day when up and walking about, but unable to lift anything, *Kali-b.*
**Thighs**, aching in sacral region, extending down thighs, China.
**Tickling**, anxious pain in sacrum, commencing with a tickling, Graph.
**Tingling**, intolerable tingling stitches in lower part of sacrum and in other places, Lyco.
**Touch**, cries out if any attempt is made to touch sacrum, *Lob.*
— bruised pain across sacrum and both hips, with sensitiveness to touch, Mag-m.
**Touched**, pustules on sacrum, very sensitive when touched, Nat-c.
— violent pain in sacrum as from a bruise, especially when being touched, Graph.
— in a spot above the sacrum, stitches on being touched, Calc-c.

SACRUM.

**Turning**, pain less when walking than when rising from a seat, turning in bed, and every motion sideways, Staph.

— when quickly turning the body there arises suddenly a dull pain in the sacrum which shows itself more while sitting or lying down through the day, Mag-m.

**Twitching,** spasmodic twitching pain from sacrum toward anus, Calc-c.

— pain in sacrum when stooping so that he could not raise himself for a time, Kali-c.

**Twisting,** severe griping and twisting in sacrum as with a pair of tongs, and then also pain in arms and feet as if it would turn them outward, Graph.

**Ulceration** of sacrum after typhoid fever, *Ars.*

**Uterus,** pain commencing in sacral region internally and apparently coming around to uterus, Syph.

**Violent** pain in sacrum which does not allow her to lie on her back, awakens her at 2 A. M., Nitrum.

— pain in sacrum, *Berb.*

— pains in sacrum, Carbo-a.

— most violent pain in sacrum after walking, Nat-c.

— stitch in region of sacrum, Bar-c.

— very violent pains in sacrum for two hours, Graph.

— pains in sacrum when and after stooping, Sarsa.

— after menses, violent pain in sacrum as if bruised during stooping and at other times in afternoon and evening, Mag-c.

**Wakes,** pains in sacrum wakes him at night, *Colch.*

**Walking,** pain in sacrum after walking or standing for a time, Kali-c.

— a broken sensation in lumbo-sacral region alternating with headache, worse by sitting, better by walking or standing, Melil.

— severe pain in sacrum after walking a little, with nausea and lassitude, Con.

— pain in sacrum in afternoon when walking, Sep.

— pain in sacrum when walking on a level, Ver-a.

### SACRUM.

**Walking,** pain in back and sacrum as if broken after walking for an hour, Plat.

— tensive pain at slightest movement extending from sacrum over hip impeding walking, Sarsa.

— severe pain in sacrum almost only when moving, so that he can hardly walk ; it seems to be in the bone, Nit-ac.

— pain in sacrum more while walking than when at rest, Mez.

— sudden violent stitch in sacrum while walking in open air, *Agari.*

— constant ache in sacrum and hips, much worse by walking or stooping forward, *Æscul.*

— backache with dragging in sacrum and stringy colorless leucorrhœa, beginning when walking, Aletris.

— pain above the sacrum while walking not when sitting, Sul.

— pain in sacrum worse by standing better by walking, Phos.

— weakness in sacrum after a walk, Petrol.

— bruised pain in sacrum while walking, Hepar.

— a pressing in sacral region only while walking and particularly setting down left foot, Spong.

— pain in sacrum less when walking than when turning in bed or rising from a seat if turning sideways, Staph.

— tensive pain in top of left sacrum while walking, Sumbul.

— sprained pain in left sacral region when walking, Sul.

— sticking and drawing in sacrum while walking, Bry.

— sticking in left sacrum while walking, Calc-c.

— drawing pain in sacrum and a sensation as if it were broken, in walking, standing and lying, Carbo-a.

— Shooting and pain in sacrum only when sitting, not when walking, Nat-c.

— pain in sacrum while walking, Borax.

— pain in sacrum drawing hither and thither, worse by walking, Hepar.

— hard pressure in sacrum, diminished while walking, Sul.

— pain in sacrum, better by walking, Mag-c.

— back and sacrum stiff, cannot be bent ; after some exertion in

SACRUM.

riding, walking and stooping, he can raise himself afterwards, only slowly and with much difficulty, Lyco.

**Weakness** in sacrum after a walk, Petrol.

— and drawing in sacrum, Sul.

— of sacro-iliac region, *Sep.*

— in sacral region from crest of ilium down outside of thighs to knees, beginning in afternoon, Nat-ars.

— in sacrum in afternoon when writing, standing at a high desk, Lith-c.

— pressure, tension and weakness in lumbar and sacral region, *Zinc.*

— pain in sacrum as from weakness after lying down in evening, Zing.

— in sacrum as if paralyzed, could neither stand or walk right, Nat-m.

— in sacrum mornings, Calad.

— and paralytic feeling at lumbo-sacral junction, *Phos.*

**Weariness**, on right side of sacrum, worse by walking, Bry.

— and pain in sacrum, Sep.

**Weight**, sensation of weight in pelvis, Gnaphal.

**Wrenched** feeling in back and sacrum with pain as from overlifting, *Cal-c.*

**Writhing**, severe drawing writhing pains in sacral region and bowels, radiating upward and downward until whole body and fingers and toes become enveloped in spasms so severe as to elicit shrieks, *Diosc.*

---

New Method of Extracting Foreign Bodies from the Nasal Fossæ in Children.—M. Felizet (*Journal des Practiciens*) related to the Surgical Society a method of extracting foreign bodies from the nose in children, which he has used with satisfactory results for five years. He injects through the sound nostril a current of warm salt water at a moderate pressure which, returning by the posterior nares of the occluded nostril, forces the foreign body out, or at least allows of its being seized with forceps.—*New York Medical Journal.*

